# Extracts from Records of Boston Society for Medical Improvement

**Published:** 1855-07

**Authors:** 


*Extracts from Records of Boston Society for Medical Improvement.*

*Intermittent Fever; Homeopathic Treatment; persistence and aggravation of the Symptoms; Treatment by Quinine and other usual means; immediate arrestation of the Chills; rapid recovery.*—Dr. MORLAND read the following account. It is well known that homœpathists adduce the curative action of cinchona in intermittent fever, as "one of the strongest possible proofs" of the truth of their doctrines; in fact, "It was in attempting to ascertain how cinchona cured ague or intermittent fever, that Hahneman made his alleged discovery." While experimenting upon himself with the bark, "an intermittent fever ensued," and he came to the conclusion that the drug must cure intermittent solely by its power, as he asserted, of producing like symptoms in a well person; and hence his deduction "*Similia similibus curantur.*"

Professor SIMPSON (*Homeopathy: its Tenets and Tendencies;* Edinburgh, 1353) has well shown the fallacy of the premises upon which Hahnemann founded his system of practice; and he has abundantly proved it not to be true that intermittent symptoms are, by any means, invariably produced, as above asserted; and not only so, but he shows that such an occurrence is a very rare and quite an exceptional thing. We can readily suppose that, if the very imagination which suggested in Hahnemann's mind the idea which serves as a motto for his system be so utterly fallacious, the attempts to *cure* intermittent fever by infinitessimal doses of quinine, would signally fail; the endeavor itself thus becomes a severe test of the practice; and even homœopathists confess their want of success. (*Vide* SIMPSON, *op. cit.* p. 248, Edin. Ed.)—How absurd, in view of such facts, do the confident assertions of the majority of homeopathic practitioners become, such as the dogma that "if any remedy be homœopathically selected, it will cure," in whatever dilution it be given?

By their own showing, cinchona and its extracts are "homœopathically selected," if administered in intermittent fever; why should they ever fail in their hands, particularly when the medicine is properly and sufficiently *diluted*, to adopt their, to us, ridiculous phraseology.

In the serious matter of compromising a patient's existence, by a do-nothing course, in a case demanding the most decided remedial interference, it is unfortunate that the public cannot be enlightened as to the responsibility incurred, and the neglect practiced, not only by the practitioner who persists in leaving unassisted Nature to struggle with an opponent sure of gaining the mastery, but also by themselves, in allowing their friends thus to be left to their fate.

With a view of illustrating, in some degree, these positions, the following account of a case has been prepared.

Mrs. ———, a young lady of delicate constitution, and for several years a resident in a tropical climate, had, during the latter part of 1853, while at the South, an attack of intermittent fever of the tertian type, and of but slight intensity; it readily yielded to quinine. She came to the North, in January, 1854, and by a fatiguing journey; being far advanced in pregnancy with her third child; fatigue and apprehension caused by accidents during the journey, nearly produced miscarriage soon after her arrival, and she suffered from weakness and cough, until the period of confinement, February 26, 1854, having completed her full term. The access of labor being somewhat sudden, a midwife was first in attendance; there was retained placenta, and profuse and exhausting flowing subsequently, which, supervening upon her previously weak condition, reduced her to an alarming state; she was perfectly bloodless in appearance; greatly emaciated; her pulse rapid and feeble.—She, however, rallied; and in from three to four weeks, went down stairs to dinner. At this time, a most unfortunate epoch for the advent of new trouble, chills, followed by fever, came on, and a regular *tertian* was declared. She was attended by a homeopathic practitioner, who administered various infinitessimal doses; and, finally, but not until the tertian had become quotidian, gave quinine in so diluted a form (stating it to be the "first dilution") that, when subsequently asked by the physicians who finally managed the case, how much he gave of the salt, he was unable to say. Under this course the patient continued to grow weaker; the chills recurred, with violence, every twenty-four hours, and generally at an early hour of the morning; after each attack the patient evidently had less and less the power of resistance. On the arrival of her husband, who had been absent during this last illness, he immediately dismissed the attending practitioner, remarking that, even to the eyes of persons unskilled in medicine, it was sufficiently evident that Nature unassisted, could not, in this case at least, do the work of cure—however possible such a result might be in a robust person.

On Tuesday, April 4, 1854, I was desired to take charge of the patient; but, in view of her then almost hopeless situation, declined to do so unless with a previous consultation; which being consented to, Dr. Bigelow, Sen., saw her with me, at about noon of the above day. There had been a severe chill about ten or twelve hours previously. Dr. Bigelow expressed strong doubts as to her recovery, taking into consideration the previous history and her present very weak and alarming condition. It was, however, resolved to give quinine in as large quantity as the system seemed likely to bear, combined with nourishment sedulously and judiciously given (the latter point had been, in good measure, attended to previously;) the administration of the remedy was immediately commenced—two grains every two hours; at a second visit, same day, P. M. I directed brisk friction with warm laudanum, just before the expected chill, with fifteen drops of the same internally; dry warmth to be afterwards employed (in place of wrapping the patient in a blanket soaked in warm water, as was done by the former attendants,) and the said friction to be kept up as long as there seemed even a tendency to chill.

April 5, 10 o'clock A. M. there had been no chill; patient expressed a sense of comfort at her escape from it. Pulse, about 110, feeble; skin natural. Perge. The same course of treatment was pursued, with the same, and with constantly better results, as regarded the patient's progress towards recovery. Much of the success attained is doubtless to be ascribed to the untiring exertion of the patient's husband in giving the quinine regularly, securing the prompt administration of food, and making the frictions with his own hands; these latter, either with warm spirit, laudanum, or with the dry hand, were resorted to on the least feeling as if of threatened chill. There was, however, no recurrence of the chills which, before the change of treatment, had been so regular of access. The quinine was continued for two weeks; for the first week in the dose above stated; during the second it was gradually diminished, and finally suspended. Citrate of quinine and iron was given pretty freely during convalescence, which was rapid, when the extreme prostration is considered. The pulse, three or four days after commencing the quinine, sank from over 100 per minute, to 96, 90 and 80; it gained strength and regularity daily; the appetite became strong; the digestion was good; color returned to the previously white lips. In the second week of treatment the patient could walk across the floor, with assistance (ten days before this she could scarcely lift her hand,) in a few days she was out, and in a very short time, she whose chance of life was pronounced "not worth the toss of a dollar," went from Boston to Washington, D. C., bore the journey well, grew stronger and gained flesh rapidly, has since gone to Europe, and by late accounts is quite well.

I firmly believe that what is said by Dr. Bartlett (Treatise on

Fevers, 1847) of the "congestive form" of periodical fever, would have proved true in this case of simple intermittent, had the same course been continued under which it became so grave. Dr. B. says: "The paroxysms must be arrested or the patient will die ; the only agent in our possession, by which this can be done, is the bark, (cinchona) and its preparations ; and no time is to be lost in their use." (Op. cit. p. 391.) The question will arise, how any well-educated practitioner (in this instance the homeopashist in attendance was such) could, in conscience, allow the disease to progress, when to his knowledge, he had the means of arresting the paroxysms at hand. One more quotation seems so apposite that it may be admitted: "All that respects the disease, and all that respects the remedy, is so marked, so sudden, and so forcible, that physicians neither doubt nor reason about the matter. They see what happens, and, resting upon the evidence of what they see, they know that the disease is cured by (mercury)" quinine. (Latham On the Heart, vol. i. pp. 266-7.) Dr. Latham is speaking of mercury—by substituting "quinine" the sentence is quite in place.

It is often remarked that one isolated case proves nothing ; granted—yet an aggregate of such cases will surely prove something ; and amidst the boasted "cures" of the homeopathists, it seems but simple justice that a counter-report should occasionally be made. To most legitimate practitioners, however, such cases, singly, must carry their own evidence.

At the next subsequent meeting (February 12th, 1855) Dr. Gould related the following case, of which he had been reminded by the above :

A gentleman from the State of New York, on a visit to Boston had an attack of tertian, and placed himself under homœopathic treatment. The paroxysms grew more severe, and became quotidian. Consultations were held, but no abatement was experienced ; and after two weeks, being very much exhausted, it was concluded by both patient and doctor, to abandon that method of treatment. On surrendering his patient, the physician remarked, that he presumed that the disease would soon be arrested, as it was well known that quinine would control fever and ague. Being asked, why then did he not employ it, he replied that it was not in accordance with their doctrine, and therefore he preferred not to try it.

On visiting the patient, he was found to be deeply jaundiced, and his liver protruding from under the ribs ; bowels constipated. Blue pill was given, and hot fomentations were applied to the hepatic region. The bowels were freely evacuated, discharging large quantities of bile. One paroxysm, only, occurred subsequently, and the recovery was very rapid and complete. No quinine was given.

[The statement of the homœopathist, in this instance, that the use of quinine in intermittent fever is "not in accordance with their doctrine," only serves to expose his ignorance of his master's teachings, and indeed of the basis of the "doctrine;" as noticed in the remarks prefatory to the first case.—*Secretary.*]—Amer. Jour. Med. Science.

